# The Use of Probiotics as Adjuvant Therapy of Periodontal Treatment: A Systematic Review and Meta-Analysis of Clinical Trials

**DOI:** 10.3390/pharmaceutics14051017

**Published:** 2022-05-09

**Authors:** Louis Hardan, Rim Bourgi, Carlos Enrique Cuevas-Suárez, Maythé Flores-Rodríguez, Arianna Omaña-Covarrubias, Marco Nicastro, Florin Lazarescu, Maciej Zarow, Paulo Monteiro, Natalia Jakubowicz, Patrycja Proc, Monika Lukomska-Szymanska

**Affiliations:** 1 Department of Restorative Dentistry, School of Dentistry, Saint-Joseph University, Beirut 1107 2180, Lebanon; louis.hardan@usj.edu.lb (L.H.); rim.bourgi@net.usj.edu.lb (R.B.); 2 Academic Area of Dentistry, Autonomous University of Hidalgo State, Circuito Ex Hacienda la Concepción S/N, San Agustín Tlaxiaca 42160, Hidalgo, Mexico; 3 Academic Area of Nutrition, Autonomous University of Hidalgo State, Circuito Ex Hacienda la Concepción S/N, San Agustín Tlaxiaca 42160, Hidalgo, Mexico; fl346930@uaeh.edu.mx (M.F.-R.); aomana@uaeh.edu.mx (A.O.-C.); 4 “Studio Nicastro” Dental Clinic, Corso Trieste 142, 00198 Roma, Italy; m.nicastro@mac.com; 5 “Trident” Dental Clinic and Postgraduate Course Centre, Str. Dr. Louis Pasteur 1A, 050533 Bucharest, Romania; florin.lazarescu@clinicatrident.ro; 6 “NZOZ SPS Dentist” Dental Clinic and Postgraduate Course Centre, pl. Inwalidow 7/5, 30-033 Cracow, Poland; dentist@dentist.com.pl (M.Z.); nljakubowicz@gmail.com (N.J.); 7 Clinical Research Unit (CRU), Centro de Investigação Interdisciplinar Egas Moniz (CiiEM), Egas Moniz, CRL, Monte de Caparica, 2829-511 Caparica, Portugal; paulojorgemonteiro@yahoo.ca; 8 Department of Pediatric Dentistry, Medical University of Lodz, Pomorska 251, 92-213 Lodz, Poland; patrycja.proc@umed.lodz.pl; 9 Department of General Dentistry, Medical University of Lodz, 251 Pomorska St., 92-213 Lodz, Poland

**Keywords:** gingivitis, lactobacillus, oral health, periodontal health, periodontal treatment outcomes, periodontitis

## Abstract

For many years, the use of probiotics in periodontitis treatment was reflected in their abilities to control the immune response of the host to the presence of pathogenic microorganisms and to upset periodontopathogens. Accordingly, the aim of the present study was to assess the use of probiotics as adjuvant therapy on clinical periodontal parameters throughout a systematic review and meta-analysis. The literature was screened, up to 4 June 2021, by two independent reviewers (L.H. and R.B.) in four electronic databases: PubMed (MedLine), ISI Web of Science, Scielo, and Scopus. Only clinical trials that report the effect of the use of probiotics as adjuvants in the treatment of periodontal disease were included. Comparisons were carried out using Review Manager Software version 5.3.5 (The Nordic Cochrane Centre, The Cochrane Collaboration, Copenhagen, Denmark). A total of 21 studies were considered for the meta-analysis. For the index plaque, the use of probiotics did not improve this clinical parameter (*p* = 0.16). On the other hand, for the periodontal pocket depth, the clinical attachment loss, the bleeding on probing, and the use of probiotics as adjuvant therapy resulted in an improvement of these parameters, since the control group achieved statistically higher values of this parameter (*p* < 0.001; *p* < 0.001; and *p* = 0.005, respectively). This study suggests that the use of probiotics led to an improvement in periodontal pocket depth, clinical attachment loss, and bleeding on probing parameters. On the other hand, this protocol seems to not be beneficial for the index plaque parameter.

## 1. Introduction

The first trigger of periodontal disease is the accumulation of dental plaque due to poor oral hygiene [1]. According to this, it is defined as inflammatory conditions that affect tissues of the teeth, which leads to the formation of pockets, gingival recession, and therefore, there is attachment loss and bone loss [2]. The etiology of periodontal disease is associated with bacterial plaque and considers three important factors that will determine whether the disease develops; these factors are: a susceptible host, the presence of pathogenic species, and the reduction or absence of beneficial bacteria [3]. Additionally, the role of other microorganisms should be highlighted, such as fungal species, which may act as a cofactor inducing the production of pro-inflammatory cytokines and favoring the occurrence of periodontal attachment loss [4,5]. Epidemiological studies show that periodontal disease and gingivitis represent a serious public health problem that can lead to systemic diseases such as diabetes and cardiovascular diseases. Consequently, the prevention and treatment of periodontitis is crucial not only for dental maintenance and oral health but also for general health [6].

Scaling and root planning is a non-surgical treatment that removes the tartar from the crown and the root surfaces of the teeth and thus leads the reduction of the microorganisms load [7]. Scaling and root planning is the treatment considered as the gold standard, and this type of treatment has been shown to reduce the bacterial load and eliminate plaque and tartar. Supportive treatments for scaling and root planning include systemic and local antibiotics, local drug delivery, host modulation therapy, lasers, and other novel methods [8].

For many years, probiotics have been used in general medicine for the treatment of inflammatory bowel diseases and vaginal infections and for the prevention of allergies and respiratory infections [9]. In dentistry, the probiotics might prevent or treat oral diseases such as caries, gingivitis, or periodontitis [10]. Commonly used probiotics in dentistry are *Bifidobacterium* and *Lactobacillus* [11]. There is evidence that the use of a probiotic yogurt supplemented with *Bifidobacterium animalis subsp. lactis* (B. lactis) could have a positive effect on plaque buildup and gingival inflammation [1]. 

Probiotics are living microorganisms. When they are directed in correct amounts, they provide a benefit for the health of the host. Therefore, the probiotics strengthen the immune system and act against allergies, stress, and toxic substances [11]. It has been revealed that the use of probiotics (*Lactobacillus reuteri*) has diminished gingival bleeding and has also led to a decrease in gingivitis. On the other hand, oral administration of the probiotic *Lactobacillus salivarius* perfected the periodontal status of healthy volunteers, especially for smokers, except non-smoking volunteers (never/ex-smokers) [12].

Although the use of probiotics seems to be beneficial, the question of whether the use of this adjunct therapy could improve the periodontal disease remains. Hence, the objective of this study is to systematically review the literature to evaluate the use of probiotics as adjuvant therapy on clinical periodontal parameters. The null hypothesis to be tested is that the use of probiotics as adjuvant therapy will not have any influence on clinical periodontal parameters. 

## 2. Materials and Methods

This systematic review and meta-analysis was performed in accordance with the PRISMA guidelines [13]. The following PICOS framework was used: population, periodontal disease; intervention, scaling, and root debridement plus the application of probiotics as adjuvant therapy; control, scaling, and root debridement only; outcome, clinical periodontal parameters; and study design, randomized clinical trials. The research question was: “Does the use of probiotics as adjuvant therapy for scaling and root debridement improve the clinical periodontal parameters?”.

### 2.1. Literature Search

The literature search was conducted by two independent reviewers up to June 04, 2021. No data limit was used for the search. Four electronic databases, PubMed (MedLine), ISI Web of Science, Scielo, and Scopus, were screened to identify manuscripts that could be included. The keywords and search strategy used in PubMed and adapted for other databases are listed in Table 1. The reviewers also performed a hand search of the reference lists of included articles for the identification of additional papers. Following the initial screening, all studies were imported into Mendeley Desktop 1.17.11 software (London, UK) to eliminate duplicates. 

### 2.2. Study Selection

Two reviewers (L.H. and R.B.) individually assessed the titles and abstracts of all studies. Manuscripts for full-text review were selected according to the following eligibility criteria: (1) reported the effect of the use of probiotics as adjuvants in the treatment of periodontal disease; (2) included a control group where only scaling and root debridement was performed; (3) measured periodontal clinical parameters; (4) presented the data in mean and standard deviation; (5) published in the English, Spanish, or Portuguese language. Case reports, pilot studies, case series, and reviews were also excluded. Full copies of all of the potentially relevant studies were assessed. Papers that seemed to meet the inclusion criteria or had insufficient data in the title and abstract to produce a clear decision were designated for full analysis. The full-text manuscripts were considered independently in duplicate by two review authors. Any discrepancy concerning the eligibility of the included studies was decided and resolved through discussion and agreement by a third reviewer (C.E.C.-S.). Only papers that satisfied the eligibility criteria listed were included.

### 2.3. Data Extraction

The data of concern from the involved studies were extracted using Microsoft Office Excel 2019 (Microsoft Corporation, Redmond, WA, USA). These data comprised the year of publication, study design, characteristics of the included patients, periodontal disease diagnosis, type of probiotics used, clinical parameters measured, follow-up, and main outcomes. If any information was missing, the corresponding authors of the included studies were notified twice via an e-mail to retrieve the missing data. If the authors did not respond within 2 weeks of the first contact, the missing information was not included.

### 2.4. Quality Assessment

The risk of bias of the selected articles was evaluated and classified according to the Cochrane risk of bias tool for randomized clinical trials [14]. They were assessed by two reviewers (R.B. and L.H.) according to the following items: selection bias (sequence generation, allocation concealment), performance and detection bias (blinding of operators or participants and personnel), bias due to incomplete data, reporting bias (selective reporting, unclear withdrawals, missing outcomes), and other bias (protocol record in CONSORT). Each domain was classified as having a low risk, unclear risk, or high risk of bias.

### 2.5. Statistical Analysis

Meta-analyses were performed using Review Manager Software version 5.3.5 (The Nordic Cochrane Centre, The Cochrane Collaboration, Copenhagen, Denmark). The analyses were carried out using the random-effects model, and pooled-effect estimates were obtained by comparing the standardized mean difference between the periodontal clinical parameters obtained from the control and experimental groups. Subgroups were built according to the follow-up time evaluated. A *p*-value <0.05 was considered statistically significant. Statistical heterogeneity of the treatment effect among studies was assessed using the Cochran Q test and the inconsistency I2 test.

## 3. Results

### 3.1. Literature Search

**Figure 1:**
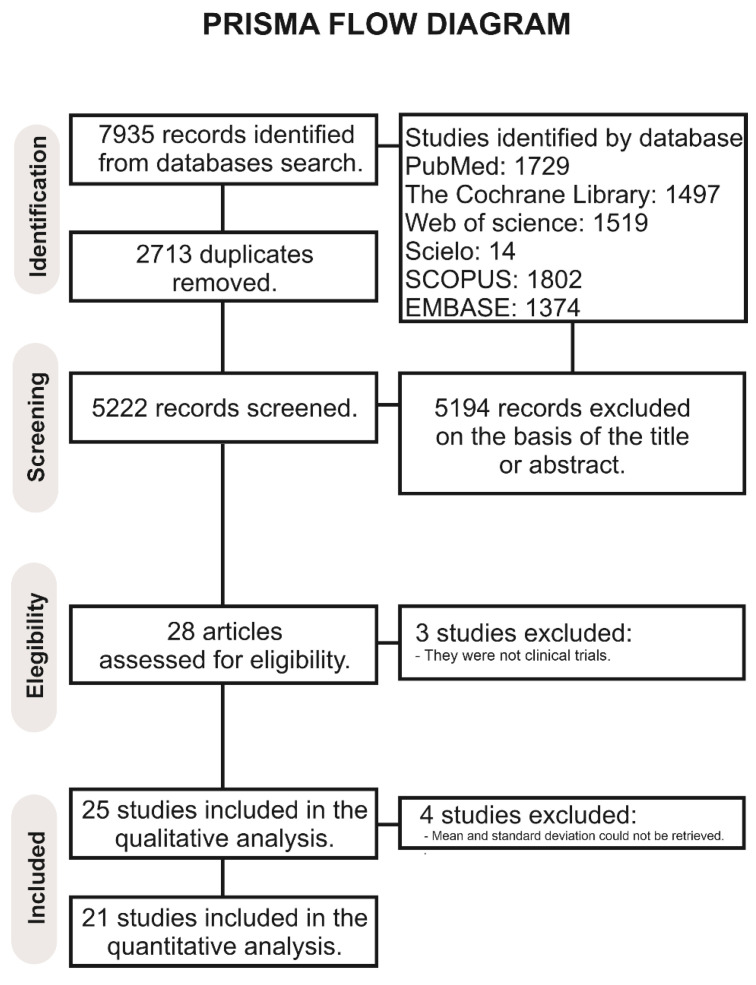
PRISMA flowchart.

The search resulted in the retrieval of 7935 records (Figure 1). After removal of duplicates, 5222 articles were screened, and 5194 were excluded based on the eligibility criteria. A total of 28 full-text articles were assessed for eligibility. Of these, 3 were not considered for the qualitative analysis because they were not clinical trials, and 25 articles were included in the qualitative analysis [1,2,3,6,7,8,9,10,11,12,15,16,17,18,19,20,21,22,23,24,25,26,27,28,29]. Of these, four studies [6,25,26,29] were excluded from the meta-analysis because the mean and standard deviation could not be retrieved. Finally, 21 studies were considered for the meta-analysis.

### 3.2. Qualitative/Descriptive Analysis

The characteristics of the studies included in the review are listed in Table 2. The studies included evaluated the performance of probiotics intake as adjuvant therapy from 4 weeks to a maximum follow-up time of 24 weeks. The probiotics tested included *Lactobacillus brevis*, *Lactobacillus plantarum*, *Lactobacillus reuteri*, *Bifidobacterium animalis*, *Weissella cibaria*, *Lactobacillus salivarius*, *Lactobacillus rhamnosus*, *Lactobacillus rhamanosus*, *Lactobacillus brevis*, *Lactobacillus plantarum*, *Lactobacillus reuteri*, and *Bifidobacterium*. Most of the included studies evaluated plaque index, bleeding on probing, probing pocket depth, and clinical attachment loss as clinical periodontal parameters.

### 3.3. Risk of Bias of the Included Studies

When analyzing the risk of bias, most studies were not at a high risk of bias except for the parameter related to reporting bias and other bias (protocol recorded at CONSORT or ClinicalTrials). The selection, performance, and detection of bias due to incomplete data were those which presented a low risk of bias (Table 3).

### 3.4. Meta-Analysis

**Figure 2:**
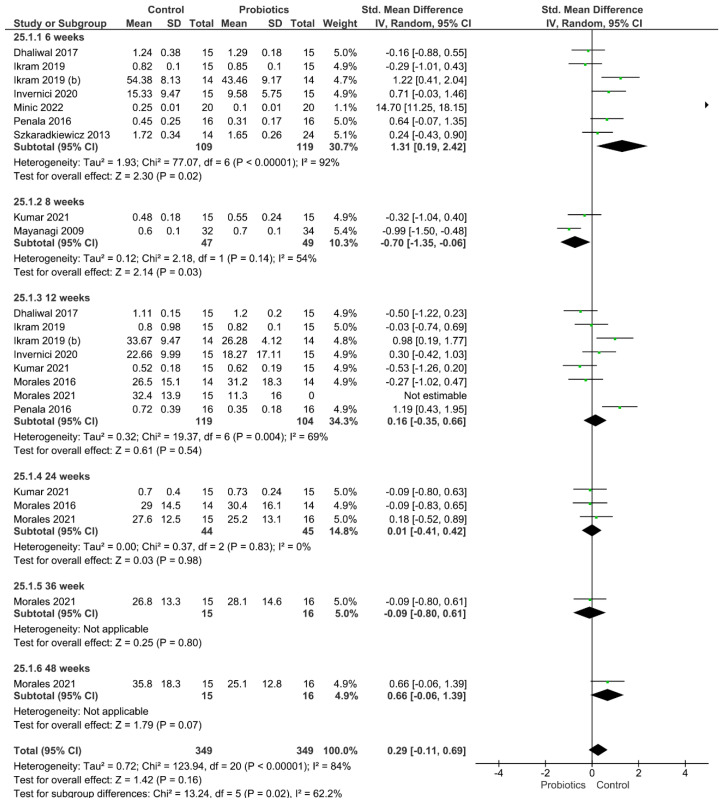
Results from the meta-analysis for the index plaque clinical parameter.

**Figure 3:**
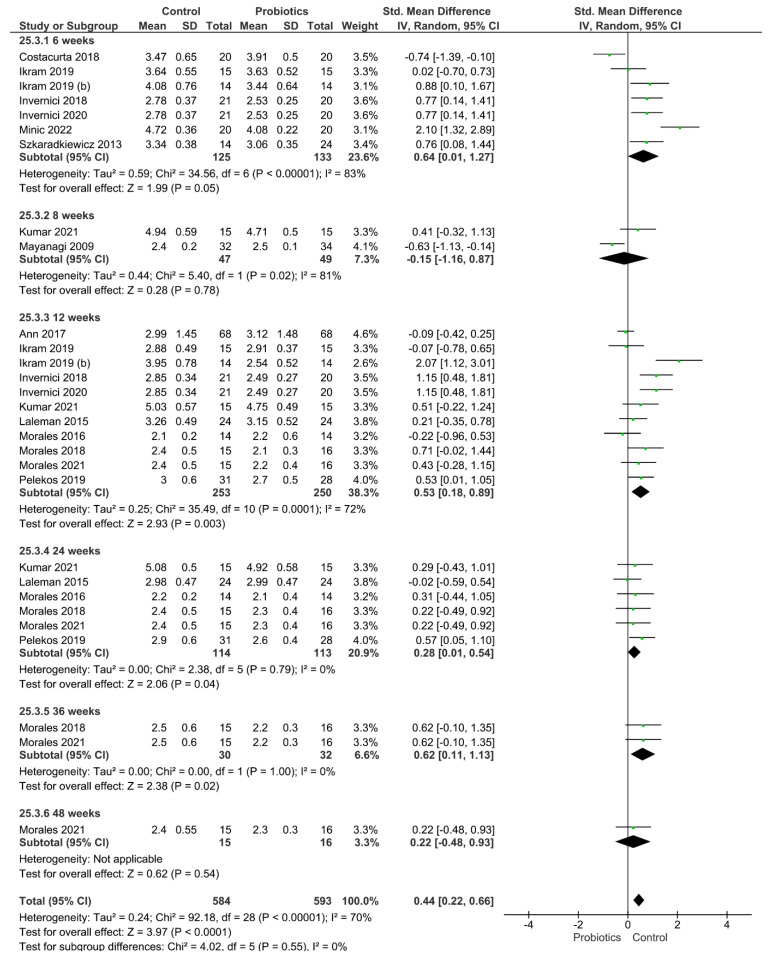
Results from the meta-analysis for the periodontal pocket depth parameter.

**Figure 4:**
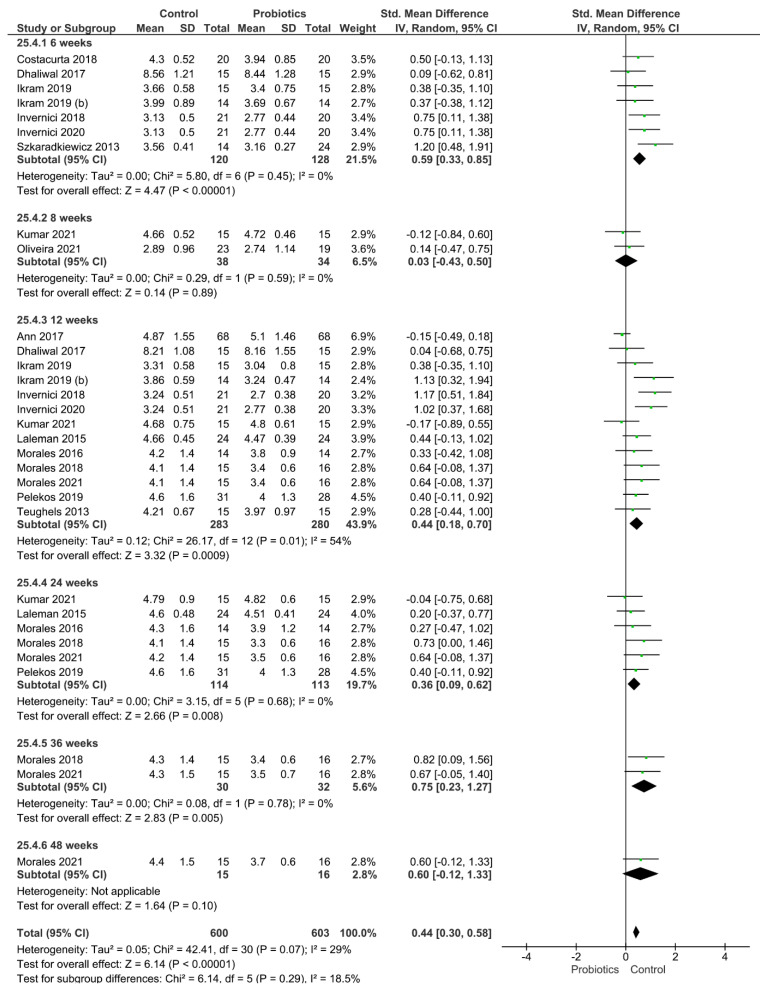
Results from the meta-analysis for the clinical attachment loss parameter.

**Figure 5:**
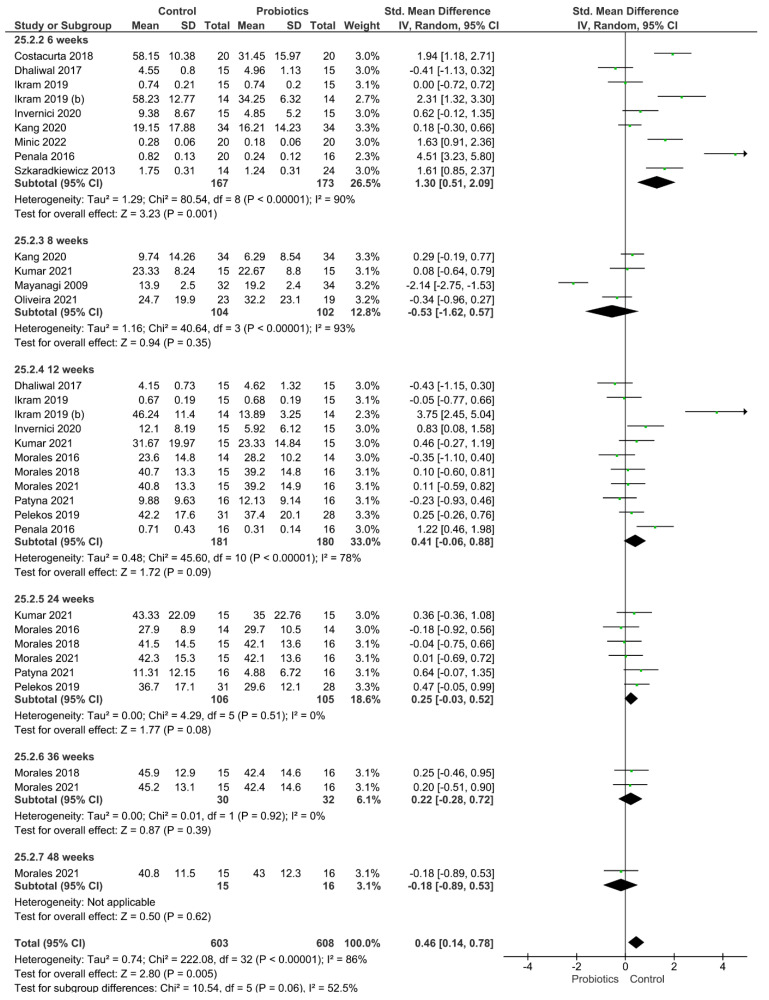
Results from the meta-analysis for the bleeding on probing parameter.

Four different clinical parameters were analyzed (Figure 2, Figure 3, Figure 4 and Figure 5). Figure 2 shows the results of the meta-analysis performed for the index plaque, where the use of probiotics did not improve this clinical parameter (*p* = 0.16). When evaluating the periodontal pocket depth, the use of probiotics as adjuvant therapy helped to improve this parameter, since the control group achieved statistically higher values of this parameter (*p* < 0.001; Figure 3). Figure 4 shows the result from the analysis of the clinical attachment loss parameter. Once again, the control group achieved statistically higher values of this parameter, meaning that the use of probiotics as adjuvant therapy resulted in an improvement (*p* < 0.001). Finally, bleeding on probing was also evaluated. The results favored the use of probiotics as adjuvant therapy, and the control group achieved statistically significant values for this clinical parameter (*p* = 0.005).

## 4. Discussion

A systematic review and meta-analysis were conducted regarding the use of probiotics as adjuvant therapy on clinical periodontal parameters. Four different clinical parameters were analyzed. For index plaque, the use of probiotics did not improve this clinical parameter, while for the periodontal pocket depth, the clinical attachment loss, and the bleeding on probing, the use of probiotics as adjuvant therapy resulted in an improvement of these parameters. Accordingly, the null hypothesis tested in this study could be partially accepted.

According to the statistical analysis, the index plaque parameter was not improved by the use of probiotics. One should bear in mind that the first initiating mechanism in dental periodontal diseases is dental plaque accumulation. This could be the result of reduced oral hygiene [30]. Thus, maintaining the gingival health might prevent the rise of gingival crevicular fluid and, subsequently, the growth of proteins that act as a source of nutrients for periodontopathogens [1]. In fact, it has been determined that one favorable approach for the treatment and control of periodontal diseases is the modulating of the host inflammatory response, as it is distinct that certain principal pathogens are fascinated by inflammation. Consequently, controlling the inflammation is of supreme significance for dealing with the infection [31]. In this situation, a potential adjuvant therapy for preventing the gingival inflammation and the dental plaque accumulation could be achieved by using probiotics [32]. These live microorganisms might suppress harmful bacteria in oral health and lead to the reduction of plaque accumulation [33,34]. This conclusion did not match with the finding of this review, as probiotics did not play a role on the improvement of the index plaque parameter. This behavior may be due to the fact that dental plaque can be reduced only with proper oral hygiene, without the need for more specialized periodontal treatment [35].

For the periodontal pocket depth, the use of probiotics as adjuvant therapy improved this parameter. Ideally, when maintaining oral hygiene and using different probiotic strains, this resulted in lessening bacterial translocation through the pocket recuperation stage [8]. Additionally, the rationale behind the use of probiotics in periodontal therapy is converting the dysbiotic pocket microbiome to a symbiotic and beneficial microbiome [36,37]. Another explanation for the improvement in the periodontal pocket depth parameter could be found in the fact that probiotics have been probed to play a protective role in the gingival epithelial barrier by maintaining protein expression, thereby preventing mucous membrane apoptosis [38]. In addition to this, the improvement of this clinical parameter should take in account the supplementary role of the presence of the probiotics through various mechanisms such as the inhibition of the growth of pathogens, the inhibition of collagenases, and the reduction of inflammation associated molecules [3].

Regarding the clinical attachment loss parameter. Once again, the control group achieved statistically higher values of this parameter, meaning that the use of probiotics as adjuvant therapy resulted in an improvement of this parameter. The clinical attachment loss is a parameter used to assess the loss of periodontal tissue support in periodontitis [39]. In this sense, it is important to explain that pro-inflammatory cytokine response plays a significant role in the nonspecific response against bacterial and fungal pathogens, and this is considered a principal mediator of periodontal disease [40]. Some reports have explained that a decrease in the levels of TNF-a, IL-1b, and IL-17 in the periodontal pockets of patients with periodontitis is observed following the treatment with the probiotic strain of *L Lactobacillus reuteri*, which may carry clinical significance [41,42]. The decrease in the pro-inflammatory cytokine response in chronic periodontitis caused by the application of probiotics may explain the results obtained in this meta-analysis.

Finally, bleeding on probing was also evaluated, and the results also favored the use of probiotics as adjuvant therapy. Bleeding on probing is a primary parameter to set the threshold for gingivitis. The insertion of a probe to the bottom of the pocket elicits bleeding if the gingiva is inflamed and if the pocket epithelium is atrophic or ulcerated, which is believed to be mediated by subgingival pathogenic microorganisms [43]. Some studies [44,45] have demonstrated that the administration of probiotics is effective in the reduction of pathogens related to the periodontal disease, which can explain the results obtained in this review. 

From this review, clinical evidence was assessed with regard to the use of probiotics as adjuvant therapy on clinical periodontal parameters. The outcomes should be considered with caution. Future research must be conducted, especially randomized controlled clinical trials, with the purpose of gaining a better understanding of the performance of probiotics in the improvement of the clinical and biochemical success of periodontal diseases. Moreover, a larger sample size with a longer follow-up should be employed in further studies. It needs to be mentioned that these findings should not be generalized, as probiotic agents were characterized by different concentrations and frequencies of application or other modes of administration. It is known that the selection of the “best” probiotic for oral health is still a controversial topic. Of interest, this study demonstrated that the use of probiotics seems to display beneficial outcomes when periodontal disease exists.

## 5. Conclusions

This study suggests that the use of probiotics leads to an improvement in some clinical parameters, including periodontal pocket depth, clinical attachment loss, and bleeding on probing. On the other hand, this protocol seems to be not beneficial for the index plaque parameter.

## Figures and Tables

**Table 1:** Keywords used in search strategy.

Search Strategy	
# 1	Periodontitis OR Gingivitis OR Periodontal therapy OR Periodontal condition OR Periodontal diseases OR Oral health OR Gingival index OR Subgingival microbiota OR Peri-implant mucositis OR Saliva microbiota OR Subgingival microbiota OR Residual pockets OR Dental plaque OR Microbiology OR Mucositis OR Periodontopathic bacteria OR Gingival crevicular fluid
# 2	Probiotics OR Lactobacillus reuteri prodentis OR Bifidobacterium lactis OR Streptococci, lozenge OR Probiotic food supplements OR Lactobacillus salivarius OR Lactobacillus brevis OR Lactobacillus plantarum OR Lactobacillus spp OR Lactobacillus rhamnosus
# 3	Randomized-controlled clinical trial OR Randomized clinical trial Double-blind method OR Randomised double-blind clinical trial OR Clinical efficacy OR Clinical evaluation OR Clinical study OR Clinical trial OR Controlled clinical trial
# 4	# 1 AND # 2 AND # 3 AND #4

**Table 2:** Qualitative analysis of the included studies.

Author and Year	Study Design	Diagnostic	Number and Age of Participants	Probiotics Used	Parameters Evaluated	Follow-Up	Main Results
Ann, 2017 [2]	Double-blinded, randomized, controlled clinical trial.	Chronic periodontitis	22 patients aged 25–45 years.	*Lactobacillus brevis*/*Lactobacillus plantarum*	Probing pocket depth Clinical attachment lost	12 weeks	There were significant decreases in pocket depths after treatment in both the experimental and control sites.
Costacurta, 2018 [11]	Randomized, controlled trial	Chronic periodontitis	40 patients aged 18–70 years.	*Lactobacillus reuteri*	Bleeding on probing Probing pocket depth Clinical attachment loss	4 weeks	The subjects with chronic periodontitis, treated with SRP and probiotics, show some beneficial effect of *Lactobacillus reuteri*, with significant reduction pf BOP and PPD.
Dhaliwal, 2017 [3]	Randomized, controlled study	Chronic periodontitis	30 patients aged 20–55 Years.	*Lactobacillus sporogenes*	Index plaque Gingival index Probing depth Relative attachment level	1 month, 45 days and 3 months	Statistically significant reductions were observed for plaque index, gingival index, and probing pocket depth, and a significant gain in the relative attachment level was observed in both groups.
Ikram, 2019 [7]	Double-blinded, randomized, controlled clinical trial.	Patients with chronic periodontitis	30 patients aged >30 years.	*Lactobacillus reuteri*	Plaque index Bleeding on probing Clinical attachment level	12 weeks	Intergroup comparison of clinical periodontal parameters did not show statistical significance.
Ikram, 2019 (b) [15]	Double-blinded, placebo controlled clinical trial.	Chronic periodontitis	28 patients aged >30 years.	*Lactobacillus reuteri*	Plaque index Bleeding on probing Probing pocket depth Clinical attachment loss	6 and 12 weeks	Intergroup comparison showed greater reduction in PPD and BOP and more gain in CAL in the probiotic group.
Ivernici, 2020 [1]	Double-blinded, randomized, controlled trial	Chronic periodontitis	30 patients aged >30 years.	*Bifidobacterium animalis subsp lactis* (HN019)	Plaque accumulation Gingival bleeding	30 and 90 days	There were no statistically significant differences between these variables. No adverse effects of probiotic therapy were observed.
Ivernici, 2018 [16]	Double-blinded, randomized, controlled trial	Chronic periodontitis	41 patients aged >30 years.	*Bifidobacterium animalis subsp lactis* (HN019)	Plaque accumulation Gingival bleeding	30 and 90 days	There were no statistically significant differences between these variables. No adverse effects of probiotic therapy were observed.
Kang, 2020 [10]	Randomized, double-blind, placebo-controlled trial	Adults without periodontitis	92 patients aged 20–39 years.	*Weissella cibaria* CMU	Bleeding on probing Probing depth Gingival index Plaque index	4 and 8 weeks	BOP improved more in the probiotic group over 8 weeks. No significant intergroup differences in PD, GI, and PI were observed during the intervention.
Kumar, 2021 [8]	Randomized, controlled clinical trial	Chronic periodontitis	48 patients aged 39–42 years.	*Lactobacillus reuteri*	Pocket depth Clinical attachment level Bleeding on probing	8, 12, and 24 weeks	At 24 weeks, the probing pocket depth and clinical attachment level improved in all groups from baseline, with no significant intergroup differences.
Laleman, 2015 [17]	Randomized, controlled trial	Severe adult periodontitis	48 patients aged 37–58 years.	*Streptococci*	Pocket probing depth Bleeding on probing Relative attachment levels	12 and 24 weeks	No significant intergroup differences could be detected at any time point
Mayanagi, 2009 [12]	Double-blinded, placebo controlled, randomized clinical trial	Periodontitis	66 patients aged 44–45 years.	*Lactobacillus salivarius* WB21	Probing pocket depth Gingival index Bleeding on probing Plaque index	4 and 8 weeks	Multivariate analysis showed that significantly higher odds were obtained for the reduction of *Tannerella forsythia* in the subgingival plaque of the test group.
Minic, 2022 [9]	Randomized prospective study	Periodontitis	80 patients age non-specified.	*Lactobacillus reuteri*	Index plaque Bleeding on probing Probing pocket depth	7 and 30 days	Topical application of probiotics in combination with SRP increases the effectiveness of conventional non-surgical therapy of periodontitis.
Morales, 2017 [18]	Randomized, placebo-controlled trial	Chronic periodontitis	47 patients aged 46–52 years.	*Lactobacillus rhamnosus*	Clinical attachment loss Probing pocket depth Bleeding on probing Plaque accumulation	3,6 and 9 months	All groups showed improvements in clinical and microbiological parameters at all time points evaluated.
Morales, 2016 [20]	Randomized clinical trial	Chronic periodontitis	28 patients aged 46–52 years.	*Lactobacillus rhamanosus* SP1	Clinical attachment loss Probing pocket depth Bleeding on probing Plaque accumulation	3 and 6 months	Both groups improved their clinical parameters.
Morales, 2021 [19]	Randomized, controlled clinical trial.	Stage III periodontitis	47 patients aged 46–52 years.	*Lactobacillus rhamnosus*	Probing pocket depth, bleeding on probing, clinical attachment loss, and plaque index.	3, 6, 9 and 12 months	The use of probiotics as an adjunct therapy failed to provide additional benefits in the treatment of stage III periodontitis.
Nedzi-Gora, 2020 [6]	Randomized intervention study	Periodontitis I and II	51 patients aged 53–55 years.	*Lactobacillus salivarius* SGL03	Index plaque Bleeding on probing	30 days	There were no changes in the PI scores between and within the groups. The value of BOP decreased in both groups.
Oliveira, 2021 [21]	Randomized, controlled clinical trial	Periodontitis	48 patients aged >18 years.	*Lactobacillus spp.* and *Bifidobacterium spp.*	probing depth and clinical attachment level	2 months	Systemic probiotics did not provide clinical or microbiological benefits in the treatment of periodontitis.
Patyna, 2021 [22]	Randomized, controlled, clinical pilot study	Periodontitis (stages II and III, grade B)	48 patients aged 57–59 years.	*Lactobacillus brevis* 7480 CECT and *Lactobacillus plantarum* 7481 CECT	Bleeding on probing Gingiva-Index simplified Plaque Control Record	3 months, and 6 months	All treatment modalities demonstrated clinical improvements in PPD and CAL at 6 months but without a statistically significant difference between the groups.
Pelekos, 2019 [23]	Double-blinded, paralleled-arm, placebo-controlled, randomized clinical trial	Periodontitis	41 patients aged 52–54 years.	*Lactobacillus reuteri*	Clinical attachment levels Probing pocket depths	90 and 180 days	Among the test and control groups, there were significant intra-group differences in primary outcomes: CAL and PPD; percentage of sites with bleeding on probing and visible plaque. There were no statistically significant inter-group differences.
Penala, 2016 [24]	Randomized, controlled trial	Chronic periodontitis.	32 patients aged 25–59 years.	*Lactobacillus* and *Bifidobacterium*	Plaque index Modified gingival index Bleeding index Probing depth (PD) Clinical attachment level	1 and 3 months	All the clinical and microbiological parameters were significantly reduced in both groups at the end of the study.
Petrushauko, 2020 [25]	Randomized clinical trial	Chronic periodontitis of I and II degrees of severity	28 patients aged 40 to 55 years.	*Lactobacillus acidophilus* and *Lactobacillus rhamnosus*,	Fedorov-Volodkina HI score, Green-Vermillion HI Score, PMA gingival index, and Mühlemann Papillary Bleeding Index.	1st, 5th and 10th days	Probiotic application for the treatment of generalized periodontitis contributed to the improvement of oral health.
Shimauchi, 2008 [26]	Randomized, double-blinded, placebo-controlled study	Periodontitis	66 patients aged 44–45 years.	*Lactobacillus salivarius* WB21	Probing pocket depth Gingival index Bleeding on probing Index plaque	4 and 8 weeks	Periodontal clinical parameters were improved in both groups after an 8-week intervention.
Szkaradkiewicz, 2013 [27]	Original article	Chronic periodontitis	38 patients aged 31–46 years.	*Lactobacillus reuteri*	Index plaque Gingival index Sulcus bleeding index Probing pocket depth Clinical attachment loss	Two weeks	We have detected an improvement of clinical indices (sulcus bleeding index (SBI), periodontal probing depth (PPD), clinical attachment level (CAL)).
Teughels, 2013 [28]	Randomized, placebo-controlled study	Chronic periodontitis	30 patients aged 45–46 years.	*Lactobacillus reuteri*	Clinical attachment loss Bleeding on probing	3,6, 9 and 12 weeks	All clinical parameters were significantly reduced in both groups.
Vivekan, 2010 [29]	Preliminary randomized clinical trial	Chronic periodontitis	30 patients aged 34–50 years.	*Lactobacilli reuteri* (Prodentis)	Index plaque Gingival index Gingival bleeding index Periodontal pocket depth Clinical attachment loss	0, 21 and 42 days	There were no significant differences in the clinical and microbiological parameters between the Prodentis and placebo groups.

**Table 3:** Qualitative synthesis for clinical trials. (*: articles excluded from the meta-analysis).

Study	Selection Bias	Performance and Detection Bias	Bias Due to Incomplete Data	Reporting Bias	Other Bias
Ann, 2017 [2]	Low Risk	Low Risk	Low Risk	High Risk	High Risk
Costacurta, 2018 [11]	Low Risk	High Risk	Low Risk	High Risk	High Risk
Dhaliwal, 2017 [3]	Low Risk	High Risk	Low Risk	High Risk	High Risk
Ikram, 2019 [7]	Low Risk	Low Risk	Low Risk	High Risk	Low Risk
Ikram, 2019 (b) [15]	Low Risk	Low Risk	Low Risk	High Risk	High Risk
Invernici, 2020 [1]	Low Risk	Low Risk	Low Risk	High Risk	Low Risk
Ivernici, 2018 [16]	Low Risk	Low Risk	Low Risk	High Risk	Low Risk
Kang, 2020 [10]	Low Risk	Low Risk	Low Risk	High Risk	Low Risk
Kumar, 2021 [8]	Low Risk	Low Risk	Low Risk	High Risk	High Risk
Laleman, 2015 [17]	Low Risk	Low Risk	Low Risk	High Risk	High Risk
Mayanagi, 2009 [12]	Low Risk	Low Risk	Low Risk	High Risk	High Risk
Minic, 2022 [9]	Low Risk	High Risk	Low Risk	High Risk	High Risk
Morales, 2018 [18]	Low Risk	Low Risk	Low Risk	High Risk	High Risk
Morales, 2016 [20]	Low Risk	Low Risk	Low Risk	High Risk	High Risk
Morales, 2021 [19]	Low Risk	Low Risk	Low Risk	Low Risk	Low Risk
Nedzi-Gora, 2020 * [6]	Low Risk	Low Risk	Low Risk	High Risk	High Risk
Oliveira, 2021 [21]	Low Risk	Low Risk	Low Risk	High Risk	Low Risk
Patyna, 2021 [22]	Low Risk	Low Risk	Low Risk	Low Risk	High Risk
Pelekos, 2019 [23]	Low Risk	Low Risk	Low Risk	Low Risk	Low Risk
Penala, 2016 [24]	Low Risk	Low Risk	Low Risk	High Risk	Low Risk
Petrushauko, 2020 * [25]	High Risk	Low Risk	Low Risk	High Risk	High Risk
Shimauchi, 2008 * [26]	Low Risk	Low Risk	Low Risk	Low Risk	High Risk
Szkaradkiewicz, 2013 [27]	High Risk	High Risk	Low Risk	High Risk	High Risk
Teughels, 2013 [28]	Low Risk	Low Risk	Low Risk	High Risk	High Risk
Vivekan, 2010 * [29]	Low Risk	Low Risk	Low Risk	High Risk	High Risk

## Data Availability

Derived data supporting the findings of this study are available from the first author (L.H.) on request.
